# Impact of FAK Expression on the Cytotoxic Effects of CIK Therapy in Triple-Negative Breast Cancer

**DOI:** 10.3390/cancers12010094

**Published:** 2019-12-30

**Authors:** Mei-Ren Pan, Cheng-Che Wu, Jung-Yu Kan, Qiao-Lin Li, Shu-Jyuan Chang, Chun-Chieh Wu, Chung-Liang Li, Fu Ou-Yang, Ming-Feng Hou, Hon-Kan Yip, Chi-Wen Luo

**Affiliations:** 1 Graduate Institute of Clinical Medicine, Kaohsiung Medical University, Kaohsiung 80756, Taiwan; mrpan@cc.kmu.edu.tw (M.-R.P.); R070059@kmu.edu.tw (Q.-L.L.); mifeho@kmu.edu.tw (M.-F.H.); 2 Drug Development and Value Creation Research Center, Kaohsiung Medical University, Kaohsiung 80756, Taiwan; 930220@kmuh.org.tw (C.-C.W.); swfuon@kmu.edu.tw (F.O.-Y.); 3 Department of Surgery, Kaohsiung Medical University Hospital, Kaohsiung 80756, Taiwan; 1000458@kmuh.org.tw (C.-C.W.); 890043@kmuh.org.tw (J.-Y.K.); 990306@kmuh.org.tw (C.-L.L.); 4 Division of Breast Surgery, Department of Surgery, Kaohsiung Medical University Hospital, Kaohsiung 80756, Taiwan; 5 Graduate Institute of Medicine, College of Medicine, Kaohsiung Medical University, Kaohsiung 80756, Taiwan; u100800001@kmu.edu.tw; 6 Department of Pathology, Kaohsiung Medical University Hospital, Kaohsiung Medical University, Kaohsiung 80756, Taiwan; 7 Division of Cardiology, Department of Internal Medicine, Kaohsiung Chang Gung Memorial Hospital, Kaohsiung 83301, Taiwan; hkyip@cgmh.org.tw; 8 Institute for Translational Research in Biomedicine, Kaohsiung Chang Gung Memorial Hospital, Kaohsiung 83301, Taiwan

**Keywords:** cytokine induced killer cells (CIK), focal adhesion kinase (FAK), apoptosis, cytotoxicity, programmed death-ligand 1 (PD-L1), triple-negative breast cancer (TNBC)

## Abstract

Triple-negative breast cancer (TNBC) is a special subtype of breast cancer in which several common diagnostic biomarkers are lost. Due to the loss of expression of receptors, treatment options for TNBC are limited. Therefore, finding safe and effective treatments for patients with TNBC is a major objective for clinicians. Previous studies suggested that cytokine-induced killer (CIK) cells may be beneficial for patients with a variety of tumor types. However, CIK therapy is not effective for all patients. In this study, we found that focal adhesion kinase (FAK), a non-receptor protein tyrosine kinase that regulates several cellular functions in different cells, has the potential to regulate tumor cells sensitized to CIK cells. Knockdown of FAK expression in TNBC cells or the treatment of TNBC cells with a FAK inhibitor followed by coculture with CIK cells increases death of TNBC cells, suggesting that FAK plays important roles in sensitizing tumor cells to CIK cells. This phenomenon could be regulated by a FAK-programmed death-ligand 1 (PD-L1)-related mechanism. Overall, our findings provide new insights into the cytotoxic effect of CIK cell therapy in TNBC treatment, and show that CIK cell therapy combined with FAK inhibitors may be a novel therapeutic strategy for patients with TNBC.

## 1. Introduction

Breast cancer is a heterogeneous disease and is one of the main causes of death among women worldwide [1,2]. Several biomarkers, including the estrogen receptor (ER), progesterone receptor (PR), human epidermal growth factor receptor 2 (HER2) and Ki-67 are routinely used to classify breast cancer subtypes in the clinic [3,4]. 

While most subtypes express these biomarkers, about 15–20% of patients with breast cancer show lost expression of these biomarkers and are classified as having triple-negative breast cancer (TNBC) [5]. Compared to other breast cancer subtypes, TNBC is more aggressive and has higher metastatic rates. Loss of ER, PR and Her2 expression could be the reason why there are fewer therapeutic drug choices for patients with TNBC. Chemotherapy was the most common treatment for these patients [6,7]. Nevertheless, many patients with TNBC develop a resistance to chemotherapies, relapse within 3–5 years after treatment, and have shorter overall survival [4,5,8,9,10,11]. Therefore, it is necessary to develop new strategies for TNBC to improve the overall survival and also to decrease the side effects of chemotherapy.

In recent years, many studies indicate that cancer immunotherapy may be another choice for cancer treatment [12,13,14,15,16,17,18,19,20,21,22]. Different to surgery, chemotherapy and radiotherapy, immunotherapy focuses on improving the anti-cancer abilities of immune cells and promoting the patient’s own immune system to fight diseases [18,20,23,24]. In general, immunotherapies involve cancer vaccines, immune checkpoint inhibitors, adoptive cellular immunotherapy and engineered T-lymphocyte-based cell therapy [25,26]. Among these immunotherapies, adoptive cellular immunotherapy could reconstitute host immunity and eliminate cancer cells through transfusing expanded and activated immune cells [15,16,27,28,29,30,31]. Several basic and clinical studies have shown that adoptive cellular immunotherapy is a feasible and safe method in treating malignant tumors [29,32,33,34]. Cytokine-induced killer (CIK) cells, a type of adoptive cellular immunotherapy, are considered to be an ideal candidate cell type for cancer immunotherapy [19]. CIK cells were induced from human peripheral blood mononuclear cells (PBMCs) by several cytokines and amplified to sufficient levels to eliminate cancer cells, while not targeting healthy cells or tissues. Recently, several basic and clinical studies have highlighted the potential of combining CIK therapy with other chemotherapies or immune therapies to gain potent, effective and durable clinical responses in several tumor types [16,20,28,29,35]. However, whether CIK could be a potential strategy for TNBC treatment remains unclear.

Focal adhesion kinase (FAK) is a non-receptor protein tyrosine kinase that regulates cell survival and several cellular functions in different cells and is usually overexpressed in many different cancer types [36,37,38,39,40,41,42]. In most cancers, FAK activation could promote cell motility in mesenchymal cells, otherwise, the inhibition of FAK could target cancer stem cells and block metastatic ability [36,43,44]. Several ongoing clinical trials involving FAK inhibitors have shown encouraging results, implying that FAK could be an attractive target for cancer therapy [45]. Our previous studies also indicated that FAK plays important roles in regulating the mechanical force and cytotoxic effects of radio/chemo treatments on different cancer cells [46,47]. In addition, several recent studies indicated that FAK could trigger immune-mediated tumor regression and modulate the immunosuppressive components of the tumor microenvironment; it also has a functional association with immune checkpoints, and regulates sensitivity to immunotherapy [48,49,50,51,52]. However, whether FAK also plays a role in regulating the cytotoxic effects of CIK therapy in TNBC cells is unknown. Therefore, our study aimed to investigate whether FAK contributes to regulate the efficacy of CIK therapy in TNBC.

## 2. Results

### 2.1. Activation and Expansion of CIK Cells

**Figure 1:**
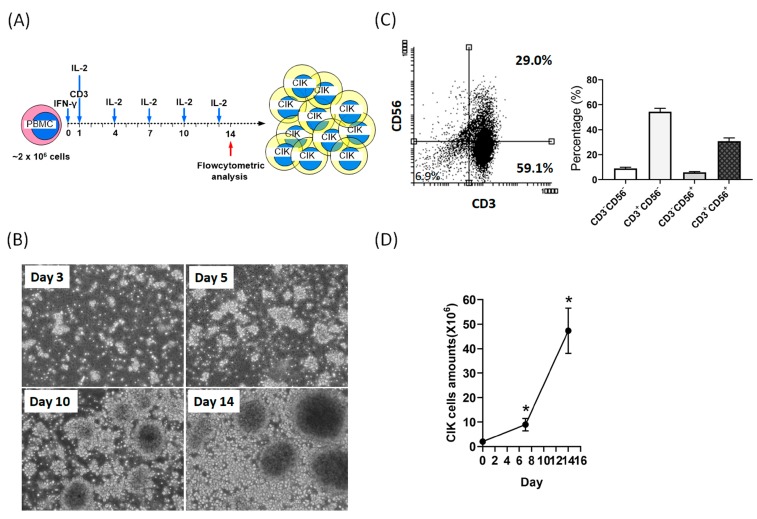
Cell preparation, morphology, expression of specific CD markers, and total amounts of cytokine-induced killer (CIK) cells. (**A**) Overview of the CIK cell preparation schedule. (**B**) CIK cells were observed under a microscope on day 3, 5, 10 and 14 after induction of peripheral blood mononuclear cells (PBMCs) (magnification, ×250). (**C**) At day 14 after induction, the cell phenotype (CD3+CD56+) was identified by flow cytometry. The mean percentage of CD3+CD56+ cells from the six donors was about 32% ± 5%. (**D**) CIK cell numbers were measured using a cell counter (LUNA automated cell counter) at the indicated time points. After 14 days of cell culture, the total number of CIK cells expanded by more than 24-fold. Data from three independent experiments were used for statistical analysis and * *p* < 0.05.

After treatment with a series of cytokines (interferon [IFN]-γ and IL-2) and anti-CD3 antibody, CIK cells derived from the PBMCs of six donors were activated and grown in suspension ex vivo. The overview of the CIK cell preparation schedule was shown in Figure 1A. Consistent with a previous study [21], we also found that activated CIK cells aggregated together to form clusters. These CIK cell clusters were first observed on the third day after induction, increased rapidly during the first seven days and continued to the fourteenth day of induction (Figure 1B). Previous studies indicated that CD3/CD56 double-positive (CD3+CD56+) cells represent activated CIK cells. 

In our study, the mean percentage of CD3+CD56+ cells after 14 days of induction was about 30% (Figure 1C). In addition, the average total amounts of CIK cells from six donors varied from 1.99 × 10^6^ to 4.73 × 10^7^ cells, which indicated a mean 24-fold expansion in our study (Figure 1D).

### 2.2. Anti-Tumor Effects of CIK Cells on MDA-MB-231 and MDA-MB-468 TNBC Cells

**Figure 2:**
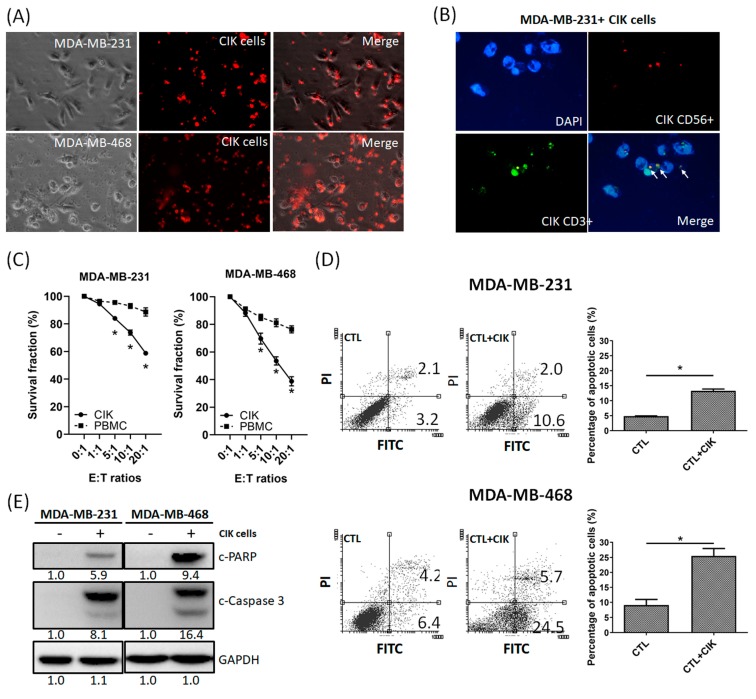
Cytotoxicity of CIK cells towards tumor cells. (**A**) Observation of the coculture of MDA-MB-231 with CIK cells (red) and MDA-MB-468 with CIK cells (red) (magnification, ×200). CIK cells adsorbed to and aggregated around the tumor cells. (**B**) Immunofluorescent (IFC) staining revealed CD3+ (green), CD56+ (red), and double-positive (CD3+CD56+) CIK cells around MDA-MB-231 cells. (**C**) Cytotoxicity of PBMCs and CIK cells against MDA-MB-231and MDA-MB-468 cells. PBMCs and CIK cells were cocultured with MDA-MB-231 and MDA-MB-468 cells at different tumor cell: CIK cell (T/C) ratios, ranging from 1:1 to 1:20 for 30 h, and were then subjected to the MTT assay. (**D**) Coculture of CIK cells with MDA-MB-231/MDA-MB-468 cells induced more cell death through apoptosis, as determined by AnV-PI double staining. (**E**) Western blot analysis showed higher PARP cleavage and Caspase-3 expression when MDA-MB-231/ MDA-MB-468 cells were cocultured with CIK cells. Data from three independent experiments were used for statistical analysis and * *p* < 0.05.

Next, we tested the anti-tumor effects of CIK cells on TNBC cells. PBMCs and CIK cells were cocultured with MDA-MB-231 and MDA-MB-468 cells at various effector to target (E:T) ratios (0:1, 1:1, 5:1, 10:1, and 20:1). Figure 2A shows CIK cells (red) cocultured with MDA-MB-231 or MDA-MB-468 cells; Figure 2B indicates that CD3+, CD56+ and CD3+CD56+ CIK cells were adsorbed and aggregated around MDA-MB-231 and MDA-MB-468 cells. After coculturing for 36 h, the suspensions were removed, and cell survival rates measured using the MTT assay. The mean percentage of MDA-MB-231 cell death after coculture with CIK cells at E:T ratios of 1:1, 5:1, 10:1, and 20:1 was 6%, 16%, 27% and 42%, respectively, and 10%, 21%, 38%, and 52% for MDA-MB-468 cells, respectively (Figure 2C). However, the mean percentage of MDA-MB-231 and MDA-MB-468 death was only about 12% and 24%, respectively, after the addition of fresh PBMCs (Figure 2C) at an E:T ratio of 20:1. Furthermore, our flow cytometric results demonstrated that MDA-MB-231 and MDA-MB-468 cells cocultured with CIK cells could significantly increase apoptotic cells at 24 h (Figure 2D). Moreover, the levels of the cleaved forms of PARP and Caspase-3 also increased under the same conditions, as determined by Western blotting (Figure 2E). 

Interestingly, the cytotoxic effect of CIK cells on MDA-MB-468 cells was stronger than that for MDA-MB-231 cells. Overall, these results indicated that CIK cells might increase apoptotic TNBC cells when cocultured with TNBC cells. 

### 2.3. FAK Inhibition of TNBC Cells Promotes the Cytotoxic Effects of CIK Cells towards TNBC Cells

**Figure 3:**
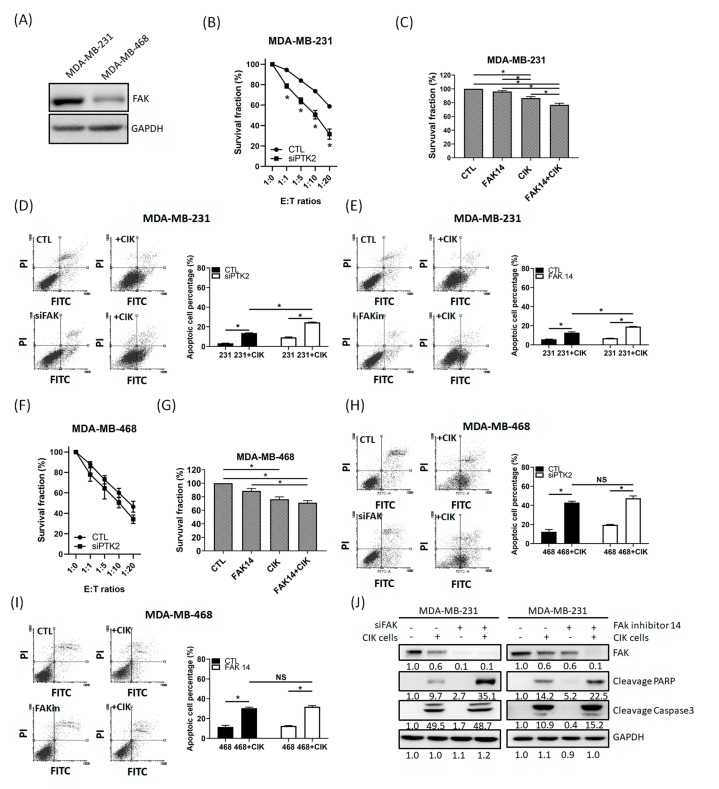
Focal adhesion kinase (FAK) inhibition in triple-negative breast cancer (TNBC) cells increased the sensitivity of TNBC cells to CIK cells. (**A**) Basal FAK expression in MDA-MB-231 and MDA-MB-468 cells. (**B**) Knockdown of FAK in MDA-MB-231 cells, followed by coculture with CIK cells increased the death of MDA-MB-231 cells. (**C**) Pretreatment of MDA-MB-231 cells with FAK inhibitor 14 (10 µM), followed by coculture with CIK cells increased the death of MDA-MB-231 cells. (**D**) AnV-PI staining indicated that knockdown of FAK in MDA-MB-231 cells, proceeded by coculture with CIK cells increased the number of apoptotic MDA-MB-231 cells. (**E**) AnV-PI staining indicated that pretreatment with FAK inhibitor in MDA-MB-231 cells, followed by coculture with CIK cells, increased the number of apoptotic MDA-MB-231 cells. (**F**) Knockdown of FAK in MDA-MB-468 cells, followed by coculture with CIK cells, increased the death of MDA-MB-468 cells. (**G**) Pretreatment of MDA-MB-468 cells with FAK inhibitor 14 (5 µM), proceeded by coculture with CIK cells, increased the death of MDA-MB-468 cells. (**H**) AnV-PI staining indicated that knockdown of FAK in MDA-MB-468 cells, followed by coculture with CIK cells, and increased the number of apoptotic MDA-MB-468 cells. (**I**) AnV-PI staining indicated that pretreatment of MDA-MB-468 cells with FAK inhibitor and coculture with CIK cells increased the number of apoptotic MDA-MB-468 cells. (**J**) Western blotting showed that PARP cleavage and Caspase-3 levels increased in FAK-depleted and FAK inhibitor-treated MDA-MB-231 cells. Data from three independent experiments were used for statistical analysis and * *p* < 0.05, NS: no significant.

A previous study suggested that FAK inhibition could cause immune-mediated tumor regression [49]. In this study, we found that the cytotoxic effects of CIK cells on MDA-MB-468 cells was stronger than that on MDA-MB-231 cells. Additionally, we found that the basal FAK expression in MDA-MB-231 cells was higher than that in MDA-MB-468 cells (Figure 3A). Therefore, we supposed that FAK expression in TNBC cells seems to play role in sensitizing the cytotoxicity of CIK cells. To identify the role of FAK in sensitizing TNBC to CIK cells, we compared the cytotoxicity induced by CIK cells in parental and FAK-depleted MDA-MB231 and MDA-MB-468 cells. 

In addition, we also treated MDA-MB-231 and MDA-MB-468 cells with low doses of FAK inhibitor 14 (10 µM) for 3 h and then cocultured them with CIK cells. We found that CIK cell coculture could promote higher cell death in FAK knockdown cells and FAK inhibitor-treated cells than in parental or untreated MDA-MB-231 cells (Figure 3B,C). Flow cytometric analysis also indicated that the percentage of apoptotic cells increased significantly more in FAK knockdown and FAK inhibitor-treated cells compared to than in parental or untreated cells (Figure 3D,E). Although these combinations also increased the cell death in MDA-MB-468 cells, there was no significant difference in cell death induced by CIK cells between parental/FAK knockdown MDA-MB-468 and FAK inhibitor-treated (+/–) MDA-MB-468 cells (Figure 3F–I). Our western bolt results also indicated that the cleavage of PARP and Caspase-3 increased in the FAK knockdown and FAK inhibitor-treated MDA-MB-231 cells (Figure 3J). We further selected another two highly metastatic TNBC cells (IV2-1 and LC-1 cells [53]) to investigate whether FAK is also sensitizing TNBC cells to CIK cells. Our results indicated that MDA-MB-231, IV2-1 and LC-1 cell have higher FAK expressions than MDA-MB-468 cells (Supplementary Figure S1A). In addition, CIK cell coculture also promoted higher cell death in FAK knockdown cells than in parental IV2-1 and LC-1 cells (Supplementary Figure S1B). Therefore, FAK might play an important role in sensitizing TNBC cells to CIK cells.

### 2.4. FAK Expression Is Correlated with PD-L1 Expression

**Figure 4:**
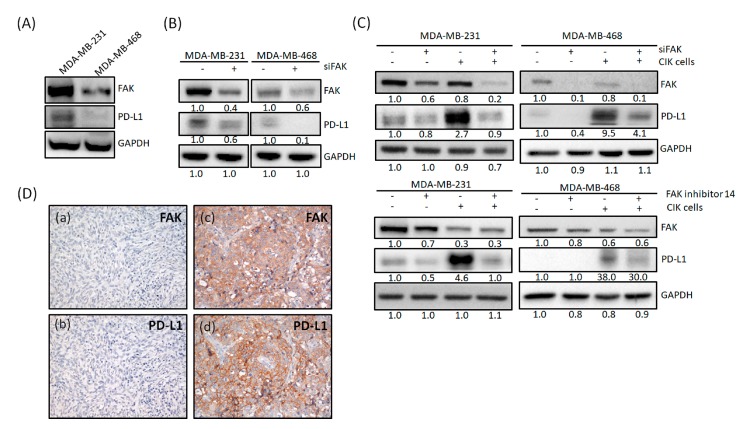
FAK expression is correlated with PD-L1 expression. (**A**) Basal FAK and PD-L1 expression in MDA-MB-231 and MDA-MB-468 cells. (**B**) PD-L1 expression decreased after siRNA-mediated knockdown of FAK expression in MDA-MB-231 and MDA-MB-468 cells. (**C**) MDA-MB-231 and MDA-MB-468 cells cocultured with CIK cells showed upregulated PD-L1 expression, but were inhibited by knockdown of FAK or FAK inhibitor treatment. (**D**) Representative immunostaining results for the expression of FAK and PD-L1 in TNBC tissues. Immunoreactivity of FAK and PD-L1 was classified as negative (**a**,**b**) or positive (**c**,**d**) based on staining observed for the cell cytoplasm and membrane. Western blot data were from three independent experiments.

Previous studies have suggested the combination of PD-L1/PD-1 blockade and CIK cells as a synergistic immunotherapy for cancer patients [54]. In addition, another study indicated that there is a correlation between FAK and PD-L1 [51]. In this study, basal FAK and PD-L1 expression were examined in MDA-MB-231 and MDA-MB-468 cells (Figure 4A). MDA-MB-231 cells showed higher expression of FAK and PD-L1 than MDA-MB-468 cells. After siRNA-mediated knockdown of FAK in both cell lines, we found that PD-L1 expression also decreased (Figure 4B). These phenomena were also seen in IV2-1 and LC-1 cells (Supplementary Figure S1C). Surprisingly, when CIK cells were cocultured with TNBC cells, the PD-L1 expression was upregulated (Figure 4C). However, if we depleted FAK or treated cells with an FAK inhibitor in MDA-MB-231 cells, this upregulation of PD-L1 could be inhibited (Figure 4C). These phenomena were also be seen in MDA-MB-468 cells (Figure 4C). Therefore, FAK seems to have the potential in regulating PD-L1 expression in TNBC cells. Further, to observe whether there is a correlation between FAK and PD-L1 in TNBC patients, we analyzed the expression of these proteins in 64 patients with TNBC. Figure 4D shows TNBC patients with either low (panel a and b) or high expression (panel c and d) of FAK and PD-L1. Consistent with our in vitro data, there was a positive correlation between FAK and PD-L1 (Table 1), suggesting that the therapeutic efficacy of CIK therapy could be potentially increased by using FAK inhibitors for TNBC treatments. 

### 2.5. PD-L1 Is Regulated through Gene Expression of FAK in TNBC Cells

**Figure 5:**
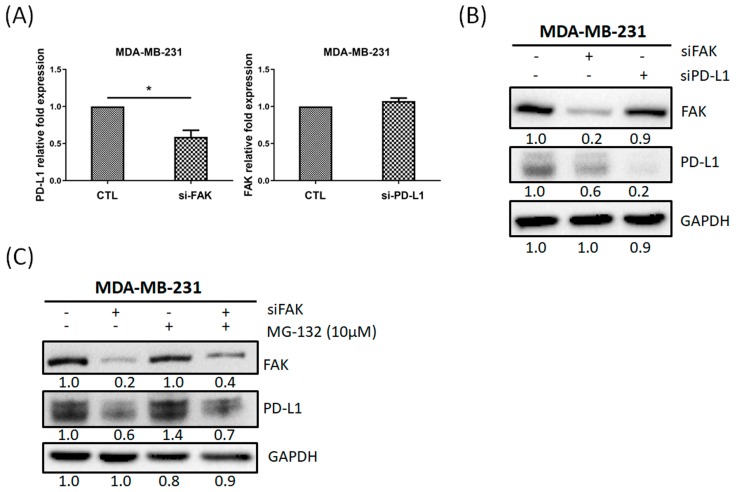
PD-L1 is regulated through gene expression of FAK in TNBC cells. (**A**) FAK inhibition decreased PD-L1 gene expression, but PD-L1 inhibition did not affect FAK gene expression in MDA-MB-231 cells. (**B**) Knockdown of FAK decreased PD-L1 protein expression, but knockdown of PD-L1 did not affect FAK protein expression. (**C**) MDA-MB-231 cells were divided into control, transfected FAK siRNA for 24 h, MG-132 (10 μM) treatment for 24 h, and transfected FAK siRNA 24 h, then combined with MG-132. Data from three independent experiments were used for statistical analysis and * *p* < 0.05.

Our in vitro and clinical results indicated that FAK might regulate PD-L1 expression in TNBC cells and there was a positive correlation between FAK and PD-L1. To investigate how FAK regulated PD-L1 expression, we first quantified the PD-L1 mRNA expression after the inhibition of FAK or PD-L1 in MDA-MB-231 cells, respectively. Figure 5A showed the PD-L1 mRNA decreased when knockdown of FAK. However, FAK mRNA did not decrease when knockdown of PD-L1. In addition, PD-L1 protein expression decreased when knockdown of FAK but FAK protein expression did not change when knockdown of PD-L1 (Figure 5B). Next, we tested whether FAK inhibition could affect the protein stability of PD-L1. Figure 5C showed that PD-L1 protein expression decreased when knockdown of FAK and increased when combined with MG-132 (a proteasome inhibitor) for 24 h. However, when knockdown of FAK for 24h was then combined with MG-132 for 24 h, this could also have recovered PD-L1 expression, but not reached the level compared with MG-132 alone. Therefore, our results suggested that PD-L1 is mainly regulated through gene expression of FAK in TNBC cells.

## 3. Discussion

Although TNBC accounts for only 15–20% of all breast cancer cases, its higher metastatic and recurrent rates, lower survival rate, and fewer therapeutic strategies, are important clinical challenges, particularly given the lack of several specific biomarkers. Therefore, to identify new targets for the diagnosis and drug development of TNBC are of critical need. 

In recent years, increasing evidence has suggested that adoptive cell therapies could be applied for cancer treatment. CIK cells, one adoptive cell therapy approach, is a group of heterogeneous immune effector cells harvested from PBMC cells that were stimulated in vitro with a variety of cytokines. Notably, these cells also show a wide MHC-unrestricted antitumor activity against several different tumor cells [55]. Several characteristics, such as rapid proliferation, fewer side effects, enhancement of anti-tumor activity, and a broader spectrum of anti-tumor activity, highlight the potential of CIK-based therapy as a treatment option for several hematologic and solid tumors, including TNBC [56,57,58]. One previous study also suggested that the biggest advantage of the infusion of CIK cells for treating malignant tumors is safety [59]. However, not all patients who receive CIK-based treatment exhibit improved outcomes. Thus, how to increase the efficacy of CIK cell therapy for treating patients with TNBC is worthy of investigation. 

In this study, CIK cell phenotypes from six healthy individuals were not evidently different, and the total amounts and quality of CIK cells were similar to that of other groups. These results demonstrate that our method for the culture of CIK cells is suitable and reproducible. In addition, the cytotoxic effect of CIK cells was stronger than that observed for PBMCs in both MDA-MB-231 and MDA-MB-468 cells, which suggests that CIK cell therapy has potential application for TNBC treatment. Several previous studies have suggested that combination with a PD-L1 inhibitor could increase the cytotoxicity of CIK cells. 

However, PD-L1 staining is variable with different PD-L1 antibodies. Therefore, it is necessary to find other druggable proteins that have a similar function as PD-L1, and could promote the cytotoxic efficacy of CIK cells.

FAK is a widely known non-receptor protein tyrosine kinase that regulates several malignant characteristics of cancer cells, including adhesion, migration, invasion, survival and proliferation [47]. A recent study suggests that FAK may regulate the inflammatory transcriptional properties that are associated with pro-tumorigenic and immunosuppressive microenvironments [48]. In addition, inhibition of FAK increases immune surveillance by overcoming the fibrotic and immunosuppressive tumor microenvironment and makes pancreatic cancer respond to immunotherapy [44,49,60]. Therefore, we first tested whether FAK deletion in TNBC cells could promote the cytotoxicity of CIK cells. Our results indicate that knockdown of FAK in both MDA-MB-231 and MDA-MB-468 TNBC cells followed by coculture with CIK cells could induce higher apoptotic cell death than that detected for parental TNBC cells cocultured with CIK cells. This increasing cytotoxic effect was also reproduced in the group treated with a low dose of FAK inhibitor, followed by coculture with CIK cells (Figure 4). These results suggest that FAK plays a role in regulating sensitivity to CIK cells in TNBC cell culture. However, there is no significant difference observed for MDA-MB-468 cells. A possible cause may be related to the low basal FAK expression in MDA-MB-468 cells; thus, knockdown of FAK in cells or FAK inhibitor treatment could only induce low levels of cell death in MDA-MB-468 cells. Our findings suggest that CIK-based cell therapy could be combined with FAK antagonist for the clinical treatment of TNBC patients.

Several small molecules inhibitors which target the FAK kinase function are currently being developed and tested in clinical trials. Many investigators have suggested that the combination of FAK inhibitor with other chemotherapies/immune checkpoint inhibitors in vitro and in vivo may be a promising approach for the cancer treatment [52]. Therefore, FAK is a promising target for anticancer therapies including immune therapy. A recent study also indicated that PD-L1 inhibitor (Atezolizumab) could collaborate with FAK to suppress cell invasion and motility in PD-L1^+^ TNBC cells [51]. Moreover, PD-L1 showed a positive correlation with FAK in The Cancer Genome Atlas (TCGA) database [51]. In this study, we found that FAK inhibition decreased PD-L1 expression (Figure 4B, Supplementary Figure S1C), suggesting that FAK might regulate PD-L1 expression. After coculturing TNBC cells with CIK cells, PD-L1 expression was upregulated in cells expressing either high or low levels of PD-L1. This upregulation could be explained as a defense from tumor cells. However, this upregulation phenomenon could be blocked by FAK inhibition (Figure 4C). Our IHC staining results also indicated that FAK was positively correlated with PD-L1 (Figure 4D). Additionally, we found that PD-L1 is mainly regulated through the gene expression of FAK in TNBC cells (Figure 5). Recent study also showed that FAK regulated PD-1/PD-L1 checkpoint signaling in a mouse model of epithelial ovarian cancer [61]. Thus, FAK might regulate TNBC cells sensitized to CIK cell therapy by regulating PD-L1. However, this FAK-PD-L1 mechanism should still be further studied by additional in vitro and in vivo studies.

## 4. Materials and Methods

### 4.1. Blood Collection

Human blood for inducing cytokine-induced killer (CIK) cells was taken from healthy donors in this study. This study was performed when obtaining the Institutional Review Board approval from Kaohsiung Medical University Hospital research ethics committee. All experimental methods were followed to the guidelines approved by the Kaohsiung Medical University Hospital (IRB: KMUHIRB-E(I)-20190097).

### 4.2. CIK Cell Preparation

After receiving informed consent, blood (10–20 mL) was collected from each healthy donor in evacuated tubes containing heparin (BD Vacutainer). Human peripheral blood mononuclear cells (PBMCs) were isolated by Ficoll-Hypaque (GE Healthcare) density-gradient centrifugation and then washed three times and plated into 25 cm^2^ flasks at a concentration of 2 × 10^6^ cells/ml in X-VIVO 15 hematopoietic medium (Lonza, 04-418Q) containing human IFN-γ (#300-02, PeproTech). On the next day, anti-CD3 monoclonal antibody (clone OKT3; 16-0037-85, eBioscience) and IL-2 (#200-02, PeproTech) were added to the medium [21]. Cells were incubated in a humidified atmosphere with 5% CO_2_ at 37 °C and changed medium every 2–3 days. After culture for at least 14 days, cells were harvested and a survival rate of >95% was observed and counted via a LUNA automated cell counter (Logos Biosystems, Inc. Annandale, VA, USA). An overview of the CIK cell preparation schedule is shown in Figure 1A.

### 4.3. Cell Lines

Human triple-negative breast cancer (TNBC) cancer cells (MDA-MB-231 and MDA-MB-468; ATCC, Rockville, MD, USA) were used in this study. In brief, cells were cultured in Dulbecco’s modified Eagle’s medium (DMEM) supplemented with 10% fetal bovine serum (FBS), 100 U/mL penicillin and 100 mg/mL streptomycin and incubated in a humidified atmosphere with 5% CO_2_ at 37 °C.

### 4.4. Flow Cytometry Analysis

Cell phenotype (CD3, CD56) was detected by flow cytometry to verify the quality of CIK cells. After 14 days of culture, CIK cells were harvested, washed twice with PBS, and then stained with CD3-fluorescein isothiocyanate (FITC) (11-0037-42, eBioscience) and CD56-PE (12-0567-42, eBioscience) antibodies and incubated for 30 min at room temperature. Prepared cells were analyzed by flow cytometry (LSR II Flow Cytometer, BD Biosciences, San Jose, CA, USA). 

### 4.5. Cell Proliferation Assay

About 1 × 10^4^ TNBC cells were seeded per well of 24-well plates and incubated for 24 h. Following treatment with different ratios of CIK cells/tumor cells (C/T) for 24–36 h, cells were washed then incubated within 3-(4,5-Dimethylthiazol-2-yl)-2,5-diphenyltetrazolium bromide (MTT) for 1–2 h. Finally, MTT was removed and the formazan crystals dissolved in dimethyl sulfoxide (DMSO), then measured at 560 nm.

### 4.6. Immunofluorescent (IFC) Staining

To distinguish CIK and tumor cells in a coculture system, PKH26 Red Fluorescent Cell Linker Kit (Merck KGaA, Darmstadt, Germany) was used to stain the CIK cells then cocultured with tumor cells. In addition, to verify CIK cell surface marker expression, we used CD3-FITC and CD56-PE antibodies to stain these markers in CIK cells. Cells were incubated with antibodies overnight at 4 °C, washed three times with PBS, and then stained with 4′,6-diamidino-2-phenylindole (DAPI). Immunofluorescent images were captured using a fluorescent microscopy. 

### 4.7. RNA Interference, FAK Inhibitor and Proteasome Inhibitor

Short interfering RNA (siRNA) against human FAK (PTK2, M-003164-02-0005), PD-L1 (CD274, L-015836-01-0005) and negative control siRNA against Firefly Luciferase (GL2) were purchased from Dharmacon Life Technologies (Cologne, Germany). Cells were transfected with 100 nM nontargeting or specific siRNA, using Lipofectamine 2000 and Opti-MEM followed the manufacturer’s protocol (Invitrogen, Carlsbad, CA, USA). FAK inhibitor 14 was purchased from Merck KGaA and dissolved in water to get stocks. MG-132 was purchased from BioVision, Inc. (Milpitas, CA, USA).

### 4.8. Quantitative Real-Time Polymerase Chain Reaction (q-PCR)

Total RNA extraction and q-PCR protocols were performed as previously described [46]. Briefly, total RNA was extracted using Trizol reagent and reverse-transcribed using SuperScript III reverse transcriptase (Invitrogen Carlsbad, CA, USA) according to the manufacturer’s protocol. Synthesized cDNA was used as a template for PCR amplification with primers for human FAK (primers forward, 5′-AGATCCTGTCTCCAGTCTAC-3′, and reverse, 5′-AATGGTTTGCACTTGAGTGA-3′), human PD-L1 (primers forward, 5′-TATGGTGGTGCCGACTACAA-3′, and reverse, 5′-TGGCTCCCAGAATTACCAAG-3′), and human GAPDH (primers forward, 5′-AAGGCTGGGGCTCATTTGC-3′, and reverse, 5′-GCTGATGATCTTGAGGCT-3′). Quantitative real-time PCR was performed in a 20-μL reaction volume using the standard protocols provided with the Roche LightCycler 480 II system (Basel, Switzerland). FAK and PD-L1 gene expression were determined as follows: ΔCT = CT (target gene) − CT (GAPDH) and ΔΔCT = ΔCT (experimental group) − ΔCT (control group).

### 4.9. Western Blotting

Antibodies against FAK (#3285), cleaved Caspase-3 (#9662S), cleaved PARP (#9541), programmed death-ligand 1 (PD-L1) (#13684), and glyceraldehyde 3-phosphate dehydrogenase (GAPDH) (#2118) were purchased from Cell Signaling Technology (Beverly, MA, USA). Antibody against PD-L1 (GTX104763) was purchased from GeneTex International Corporation (Irvine, CA, USA). (All original Western Blot figures can be found in the Supplementary file).

### 4.10. Human Specimens

In brief, tissue blocks from 64 patients with TNBC were collected from the Department of Pathology, Kaohsiung Medical University Hospital, Taiwan. Institutional Review Board approval was obtained from the Research Ethics Committee of the Kaohsiung Medical University Hospital (IRB: KMUHIRB-E(II)-20190119). Data were analyzed anonymously, so additional informed consent information was not required. All experimental methods were followed to the guidelines approved by the Kaohsiung Medical University Hospital.

### 4.11. Statistical Analysis

Statistical differences between control and experimental groups were compared by two-tailed Student’s t-test, and a *p* value < 0.05 was considered as statistically significant. All statistical data were analyzed using the SPSS software (19.0, IBM Corp., Armonk, NY, USA).

## 5. Conclusions

In conclusion, our findings provide new insights into the cytotoxic effect of CIK cell therapy in TNBC treatment. FAK plays important roles in sensitizing tumor cells to CIK cells. A possible mechanism explaining the enhanced cytotoxicity of CIK cells for TNBC cells could be related to the FAK-PD-L1 axis. CIK cell therapy combined with FAK inhibitors may be a novel therapeutic strategy for patients with TNBC.

## Figures and Tables

**Table 1:** Association of FAK and PD-L1 expressions in TNBC tissues.

FAK	PD-L1, *n* (%)		*p*-Value
	Positive (>1%)	Negative	
**High**	17 (26.5)	20 (31.3)	
**Low**	5 (7.8)	22 (34.4)	*p* = 0.047

## Supplementary Materials

The following are available online at https://www.mdpi.com/2072-6694/12/1/94/s1, Figure S1: The role of FAK in regulating TNBC cells to CIK cell.
