# Correction: Pan et al. Impact of FAK Expression on the Cytotoxic Effects of CIK Therapy in Triple-Negative Breast Cancer. *Cancers* 2020, *12*, 94

**DOI:** 10.3390/cancers13215401

**Published:** 2021-10-28

**Authors:** Mei-Ren Pan, Cheng-Che Wu, Jung-Yu Kan, Qiao-Lin Li, Shu-Jyuan Chang, Chun-Chieh Wu, Chung-Liang Li, Fu Ou-Yang, Ming-Feng Hou, Hon-Kan Yip, Chi-Wen Luo

**Affiliations:** 1 Graduate Institute of Clinical Medicine, Kaohsiung Medical University, Kaohsiung 80756, Taiwan; mrpan@cc.kmu.edu.tw (M.-R.P.); R070059@kmu.edu.tw (Q.-L.L.); mifeho@kmu.edu.tw (M.-F.H.); 2 Drug Development and Value Creation Research Center, Kaohsiung Medical University, Kaohsiung 80756, Taiwan; 930220@kmuh.org.tw (C.-C.W.); swfuon@kmu.edu.tw (F.O.-Y.); 3 Department of Surgery, Kaohsiung Medical University Hospital, Kaohsiung 80756, Taiwan; 1000458@kmuh.org.tw (C.-C.W.); 890043@kmuh.org.tw (J.-Y.K.); 990306@kmuh.org.tw (C.-L.L.); 4 Division of Breast Surgery, Department of Surgery, Kaohsiung Medical University Hospital, Kaohsiung 80756, Taiwan; 5 Graduate Institute of Medicine, College of Medicine, Kaohsiung Medical University, Kaohsiung 80756, Taiwan; u100800001@kmu.edu.tw; 6 Department of Pathology, Kaohsiung Medical University Hospital, Kaohsiung Medical University, Kaohsiung 80756, Taiwan; 7 Division of Cardiology, Department of Internal Medicine, Kaohsiung Chang Gung Memorial Hospital, Kaohsiung 83301, Taiwan; hkyip@cgmh.org.tw; 8 Institute for Translational Research in Biomedicine, Kaohsiung Chang Gung Memorial Hospital, Kaohsiung 83301, Taiwan

In the original article [1], there was a mistake in Figure 4C as published. Figure 4C (upper-right figure) was misplaced with our other data. For this reason, this figure should be replaced with the correct one as listed below. The quantitative results provided are also corrected.


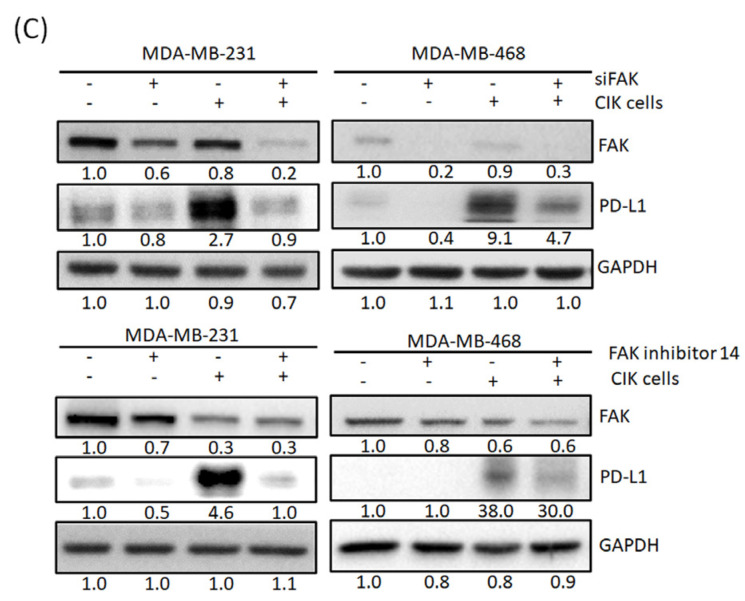


In the original article, there was a mistake in Table 1 as published. The *p*-value in Table 1 should be corrected to 0.023.

The authors apologize for any inconvenience caused and state that the scientific conclusions are unaffected. The original article has been updated.
