# Supplementary material for: Impact of FAK Expression on the Cytotoxic Effects of CIK Therapy in Triple-Negative Breast Cancer
Source: Cancers (Basel). 2019 Dec 30;12(1):94. doi: 10.3390/cancers12010094 (PMC7017032; doi:10.3390/cancers12010094)

Article

Impact of FAK Expression on the Cytotoxic Effects of CIK Therapy in Triple-Negative Breast Cancer

Mei-Ren Pan [1,2,†], Cheng-Che Wu [3,4,†], Jung-Yu Kan [3,4], Qiao-Lin Li [1], Shu-Jyuan Chang [5], Chun-Chieh Wu [2,6], Chung-Liang Li [3,4], Fu Ou-Yang [2,3,4], Ming-Feng Hou [1–5], Hon-Kan Yip [7,8] and Chi-Wen Luo [2,3,4,*]

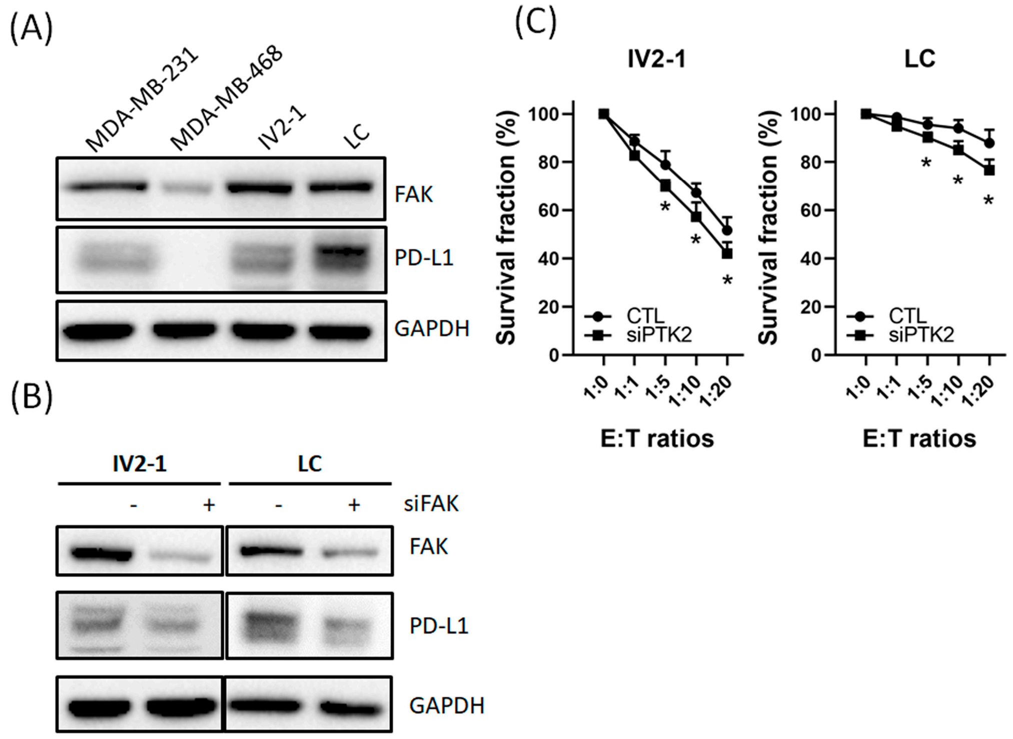

Supplementary Figure S1. The role of FAK in regulating TNBC cells to CIK cell. (**A**) Basal FAK and PD-L1 expressions in MDA-MB-231, MDA-MB-468, IV2-1 and LC-1 cells. (**B**) PD-L1 expression decreased after siRNA-mediated knockdown of FAK expression in IV2-1 and LC-1 cells. (**C**) Knockdown of FAK in IV2-1 and LC-1 cells, followed by coculture with CIK cells increased the death of both cells. Data from three independent experiments were used for statistical analysis and * $p < 0.05$.

Original Western Blot Figures

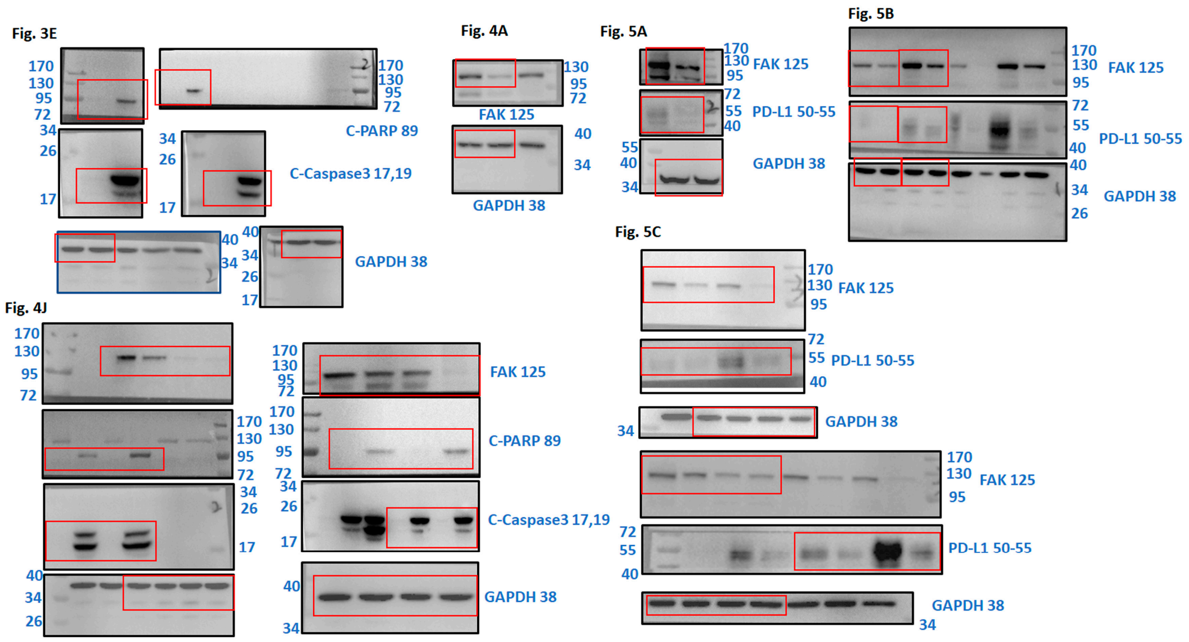

Supplement: Supplementary file 1 [file cancers-12-00094-s001.pdf]
